# Slow and population specific evolutionary response to a warming environment

**DOI:** 10.1038/s41598-023-36273-3

**Published:** 2023-06-15

**Authors:** Marta A. Santos, Marta A. Antunes, Afonso Grandela, Ana S. Quina, Mauro Santos, Margarida Matos, Pedro Simões

**Affiliations:** 1 cE3c–Centre for Ecology, Evolution and Environmental Changes & CHANGE–Global Change and Sustainability Institute, Lisbon, Portugal; 2 Departamento de Biologia Animal, Faculdade de Ciências, Universidade de Lisboa, Lisbon, Portugal; 3 CESAM–Centre for Environmental and Marine Studies, Universidade de Aveiro, Aveiro, Portugal; 4 Departament de Genètica i de Microbiologia, Grup de Genòmica, Bioinformàtica i Biologia Evolutiva (GBBE), Universitat Autònoma de Barcelona, Barcelona, Spain

**Keywords:** Evolution, Experimental evolution, Ecology, Evolutionary ecology

## Abstract

Adaptation to increasingly warmer environments may be critical to avoid extinction. Whether and how these adaptive responses can arise is under debate. Though several studies have tackled evolutionary responses under different thermal selective regimes, very few have specifically addressed the underlying patterns of thermal adaptation under scenarios of progressive warming conditions. Also, considering how much past history affects such evolutionary response is critical. Here, we report a long-term experimental evolution study addressing the adaptive response of *Drosophila subobscura* populations with distinct biogeographical history to two thermal regimes. Our results showed clear differences between the historically differentiated populations, with adaptation to the warming conditions only evident in the low latitude populations. Furthermore, this adaptation was only detected after more than 30 generations of thermal evolution. Our findings show some evolutionary potential of *Drosophila* populations to respond to a warming environment, but the response was slow and population specific, emphasizing limitations to the ability of ectotherms to adapt to rapid thermal shifts.

## Introduction

Global warming is severely impacting biodiversity worldwide^1,2^. Genetic adaptation and plasticity can help population persistence under increasingly stressful environments, but there is a limit to a sustainable rate of evolutionary change^3–5^. Recent evidence suggests that genetic adaptation to global warming might indeed be limited^6,7^. Plasticity also seems to provide limited benefit to buffer organisms from extreme temperatures^8,9^.

Evolutionary adaptive changes can be assessed by comparing the performance of populations that have evolved in different environments. In ectotherms, adaptation to different thermal environments in nature has been inferred by measuring performance in a range of temperatures under controlled conditions in the laboratory (e.g.,^10–12^). Considering the relevance of genotype-by-environment interactions in thermal responses^6,7^, it is critical to understand if and how locally adaptive responses arise in face of rapid climate warming and whether they are expressed in different environmental settings. One important caveat of most thermal adaptation studies using recently founded populations from the wild is that they do not measure performance in the specific environments where the populations have evolved, a condition that can be achieved through experimental evolution^13,14^.

Experimental evolution is a powerful tool to study adaptive responses, as it allows to apply specific selection protocols and measure adaptive response by comparing the performance of ancestral (control) and experimentally evolved populations under clearly defined environments^13,15,16^. This approach was already used in the 90s to tackle thermal evolution with evidence for substantial genetic variation for heat and cold resistance (see^17^ for a review). In the past decade, this approach has been increasingly applied in ectotherms to address adaptation to different ecologically relevant thermal scenarios^14,18–22^. Some studies have focused on evolution under increasingly warming environments^14,18,20^, with evidence for reduced evolutionary potential to respond to rapid thermal shifts. However, some important factors have been missing from such studies, namely the effect of inter-population variation in the evolutionary response and the impact of increasing thermal amplitude across generations. Furthermore, we still hold very limited understanding on the limits and pace of adaptation in face of a warming environment and whether such adaptation involves performance costs in other environments.

*Drosophila subobscura* is an ectothermic species particularly suited to study thermal adaptation because it has an ample geographical variation in nature and inversion polymorphisms that clearly respond to thermal shifts in the environment^23,24^. The range of viable development temperatures for this species is 6–26 °C^25,26^ with the optimum between 16 and 20 °C^27^. Evidence for adaptive and plastic responses to temperature have been shown in this species for both life history (e.g.^28–31^) and physiological traits^27,32,33^.

A recent experimental evolution study in two historically differentiated populations of *D. subobscura* (from contrasting European latitudes, Portugal and The Netherlands) addressed the thermal response to ecologically relevant environmental scenarios of warming environments, including selective regimes comprising thermal variation and increasing temperatures across generations^34,35^. No evidence for an adaptive response to warming conditions was found after 9 generations of evolution under such varying thermal environment. Nevertheless, by generation 31 an improved thermal performance at high temperatures occurred, but only for the populations of higher latitude^34,35^.

These experiments might have captured only a fraction of the adaptive response as the reproductive performance was tested under constant temperatures, not mimicking the dynamic thermal environments in which populations evolved. This might limit the ability to detect adaptive changes particularly if fine-tuned trade-offs are involved. Here, we use an orthogonal design to test for adaptation to warming conditions in those populations during around 40 generations of thermal evolution. The populations were first subjected to an increasingly warmer environment during around 20 generations and were thereafter maintained in a hot fluctuating regime, steady across generations. To assess shorter and longer-term evolutionary response, populations were analyzed after 22 and 39 generations of thermal selection. We expect that populations evolving under warming environments will have a better performance under those conditions than populations from which they derived (representing their ancestral state). Furthermore, provided sufficient genetic variation we expect a short-term evolutionary response (after 22 generations) to the progressively warming environment, with a lower response in the subsequent evolutionary period in which a hot fluctuating regime was maintained across generations. We also test for evolutionary trade-offs between environments, which will be expected if there are costs associated with adaptation to different environments (‘local adaptation’ sensu^15^). These experiments were done in the two populations of contrasting past history mentioned above, at two different time points of thermal evolution. The goals of this study are to: (1) address if and how fast adaptation to a warming environment occurred; (2) test whether adaptation to warming conditions entails costs (i.e. evolutionary trade-offs) in the ancestral environment; (3) analyze whether the signature of historically differentiated genetic backgrounds affects thermal evolutionary response.

## Material and methods

### Population maintenance and thermal selection regimes

Experimental populations used in this study were derived from two collections of natural populations of *Drosophila subobscura* in 2013. These collections were done in Adraga, Portugal (38º48′ N) and Groningen, The Netherlands (53º13′ N)*,* giving rise to the PT and NL populations. The number of founder females was 213 for the Adraga collection and 170 for the Groningen collection. These populations were three-fold replicated by the fourth generation^36^. Populations were maintained in vials (in general 24 for both the adult and developmental stages) with discrete generations, 12L:12D photoperiod with constant 18 °C temperature, controlled densities in eggs (70 eggs per vial) and adults (40 adults per vial), and reproduction close to peak fecundity (seven to 10 days-old flies). After 70 generations in these conditions, a warming regime was generated from each population (Warming, WNL_1-3_ and WPT_1-3_ populations)^34^. It has a daily fluctuation profile that ranged from 15 and 21 °C and increased every generation its daily mean temperature (by 0.18 °C) and amplitude (by 0.54 °C, with a lower minimal and higher maximal daily temperature). The rate of mean increase fits the expected pace of global warming increase per decade (0.19–0.63 °C, see^1^), whereas the ratio of increase in thermal extremes (amplitude) to thermal mean per generation (0.54:0.18) is also quite comparable to IPCC predictions (2:1). The increase in temperature of the warming regime led to a progressively shorter development time and consequently a reduction in the length of the life cycle, from 28 days in the controls to 24 days in the warming regime (in both latitudinal populations) by the end of the study. The adult age of reproduction in the warming regime was maintained as in the controls. This discrepancy in life cycle length led to a total number of 39 generations in the warming regime vs. 36 generations in the controls during the temporal span of this study. Aside from this decoupling generated by the different thermal profiles, all experimental populations were subjected to the maintenance protocol described above. PT and NL populations, representing the ancestral state of the WPT and WNL populations, were included in all assays as the Control populations.

At generation 22 the peak temperature experienced by the W populations reached 30.2 °C, becoming particularly stressfull and leading to very high juvenile mortality. This originated a significant crash in adult census sizes for all warming populations in that generation (a mean census size of around 200 individuals across all warming populations, see Supplementary Table 1). We estimated a juvenile mortality of around 95% at this generation, considering the total number of emerged individuals from the egg collection effort. At generation 24 there was an additional population crash with an estimated 93% of juvenile mortality (see Table S1). Because of this, the cycle was stopped and reversed to that of generation 20 with a mean temperature of 21.4 °C and fluctuating from 13.5 to 29.4 °C. This cycle then remained unaltered thereafter till the end of the experiment (from generation 23 to generation 39) — see thermal cycles in Table S2. This cycle was chosen to assure that populations were maintained under thermal selection but avoiding the risk of extinction. Apart from the population crashes referred in particular generations that led to census sizes below 300 individuals, these were generally high (between 600 and 1000 individuals) throughout most of the study (see Table S1). In the warming populations, we estimate around 75–80% mortality in the subsequent generations following the period of intense stress (between generations 25 and 32) and around 65–70% mortality between generation 33 and 39. As for the controls, estimated mortality was around 10–20% throughout the study.

### Experimental assays

Two thermal adaptation assays were done when the Warming populations were at generations 22 and 39. In an orthogonal design, Warming and Control populations were tested in their own and the other populations environment. As the Warming populations have been maintained in the same temperature cycle of generation 20 (see above), this was the Warming environment that was used in both assays, while the other environment was a constant temperature of 18 °C.

Twenty pairs of females and males were assayed from each replicate population per environment (Warming or Control), which also developed in the environment where they were assayed. In each assay, 480 pairs of flies were analysed (20 pairs × 3 replicate populations × 2 selection regimes × 2 historically differentiated populations × 2 assay environments). Reproduction was followed during the first 9 days after emergence. We characterized reproductive success by counting the number of offspring derived from day 9th eggs, emerging during a 10-day period since the first day of emergence. Three other life-history traits were estimated: age of first reproduction (number of days until first egg laying), fecundity (eggs laid between days 6 and 9) and juvenile viability (ratio between reproductive success and number of eggs laid in the 9th day). The interval between days 6 and 9 of adult life was chosen to measure fecundity as it closely matches the age of individuals that contribute to the next generation during population maintenance (which is ~ 6 to 10 days of age, when egg collection takes place for the next generation). We are thus assaying our populations at the age interval when selective pressures are expectedly higher. To minimize maternal environmental effects, assayed populations were maintained in the control environment for one full generation prior to assays.

### Statistical methods

Data from each mating pair per population and environment was used as raw data. Linear mixed effects models were applied to the data, defining a “sum to zero” contrast option for each factor. Considering the high deviations from normality assumptions, generalized linear mixed-effects models (GLMM) were applied to reproductive success, age of first reproduction, and fecundity. Models assuming different distributions—poisson, quasipoisson and negative binomial were tested and those with lowest values of Akaike information criterion (AIC) were chosen. The best models were the following: reproductive success and fecundity—quasipoisson distribution accounting for zero inflation; age of first reproduction—poisson distribution. Maximum Likelihood was the (default) estimation algorithm and Type III Wald chisquare tests were applied for significance testing. For juvenile viability data, general linear mixed effects models (GLM, gaussian distribution) were fitted with the default estimation logarithm (REML—restricted maximum likelihood) after arcsine transformation to meet normality assumptions. ANOVAs (Type III Wald F tests, Kenward-Roger degrees of freedom) were performed to estimate significance levels of differences between factors for this trait. To account for variation due to fecundity in the viability estimates, we used fecundity of the ninth day as a covariate in the viability data analysis.

Analyses were performed in R v4.0.4, with lme4^37^, car^38^, lawstat^39^, emmeans and ggplot2^40^ packages.

Specifically, we applied the following models to all traits at each generation (for simplicity we do not include the interactions with random factors but they were also included):

1 $$\begin{aligned} Y = & \mu + History + AP\left\{ {History} \right\} + Env + Selection \\ & + History \times Env + History \times Selection + Selection\quad \\ & \times Env + Selection \times History \times Env + \varepsilon \\ \end{aligned}$$

2 $$\begin{aligned} Y = & \mu + History + Env + Selection + Block \\ & + History \times Env + History \times Selection + Selection \quad \\ & \times Env + Selection \times History \times Env + \varepsilon \\ \end{aligned}$$

In model (1), *AP{History}* is a random factor, representing the Ancestral population nested in the fixed factor History (e.g. PT1 is the ancestral population of PT1 and WPT1, nested in PT origin, etc.). In model (2), *Block* is a random effect, defined as the set of same-numbered replicate populations that were assayed in the same pseudo-randomized experimental rack. *Y* is the trait reproductive success, age of first reproduction, fecundity or juvenile viability. Selection is a fixed factor corresponding to the two selection regimes (Warming and Control) and the Env is the fixed factor Environment, with two categories (Warming and Control Environments), all other terms being the interactions between fixed factors. We selected model (1) for all traits based on AIC values.

A significant interaction between selection regime and environment, with higher performance of Warming populations in the warming environment is indicative of a pattern of adaptation to warming conditions. Costs of adaptation will involve a lower performance of Warming populations than controls in the control (ancestral) environment.

To complement the statistical analyses detailed above, we measured the amount of differentiation between thermal regimes in each environment by applying the Cohen’s *d* statistic^41^.

## Results

### Reproductive success

**Figure 1:**
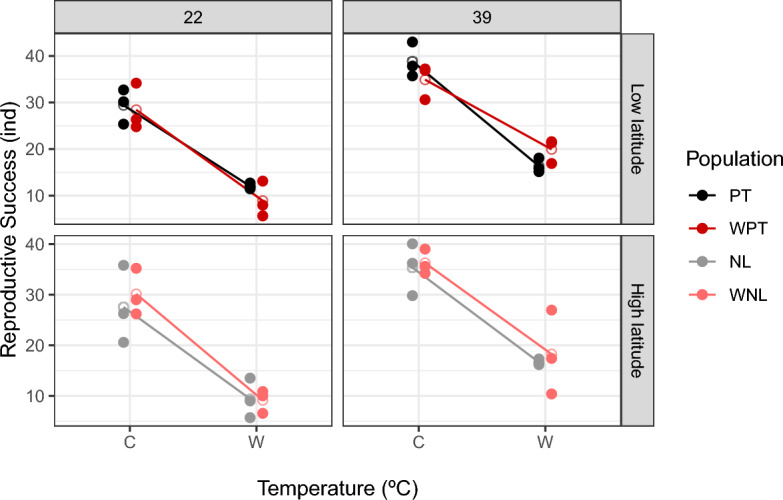
Reproductive success of the warming (WNL - warming, high latitude; WPT - warming, low latitude) and control (NL - control, high latitude; PT - control, low latitude) populations in Warming and Control thermal environments at generations 22 and 39.

At generation 22, a significant lower reproductive success was found in populations assayed in the Warming environment (Env factor: χ^2^ (d.f. = 1) = 186.5, p < 0.001; see Table S3, Fig. 1). There were no significant differences between thermal selection regimes, histories or their interactions—see Table S3.

By generation 39 a significant History × Selection × Environment interaction was observed (χ^2^ (d.f. = 1) = 3.851, p < 0.05; see Table S4 and Fig. 1), indicating differences between historically differentiated populations in how they changed thermal reaction norms due to thermal selection (see below). Also, a significantly lower performance in the Warming environment was observed, as found in generation 22 (Env factor: χ^2^ (d.f. = 1) = 163.1, p < 0.001; see Table S4 and Fig. 1).

Considering the significant interaction at generation 39 reported above, analyses were performed separately for the populations of different history. For the low latitude populations (while not for the high latitude ones) a significant interaction between selection and environment was observed (significant Selection × Environment; see Table 1 and Fig. 1). This pattern corresponds to a higher performance of the Warming populations relative to their controls in their own (warming) environment, and the reverse in the control environment (see Fig. 1).

**Table 1:** Patterns of adaptation to a warming environment for reproductive success in low latitude and high latitude *D. subobscura* populations after 39 generations of thermal selection.

	Model parameters	χ^2^ (d.f. = 1)
Low latitude populations	Selection	1.718 n.s.
	Environment	110.84*
	Selection*environment	11.627*
High latitude populations	Selection	0.159 n.s.
	Environment	62.986*
	Selection*environment	0.215 n.s.

The χ^2^ statistic and associated degrees of freedom are presented. Significance levels for χ^2^ values: p > 0.05 n.s.; p < 0.001*.

Effect sizes for reproductive success were estimated as Cohen’s *d* to have a comparative measure of the differentiation obtained between thermal regimes in each environment by generation 39 (see Table S5). For the low latitude populations, these estimates indicated a “very large” magnitude of differentiation between warming and control populations in the warming environment (Cohen’s *d* of 1.67, Warming > Control populations) corresponding to an average increase of 9.7% in performance of warming relative to control populations. A lower, albeit still “large” differentiation, was observed between these populations in the control environment (Cohen’s *d* of 1.06, Control > Warming populations). For the high latitude populations the magnitude of the differentiation was “small”, both in the warming and control environment (Cohen’s *d* below 0.27 in both comparisons). These results generally concur with those obtained from the statistical models presented above.

### Other life-history traits

Differences between environments were generally observed for all traits (age of first reproduction, fecundity and juvenile viability) in both generations, with consistent higher performance in the control environment (see Tables S3 and S4, Figs. S1–S3). No other factors or their interactions were significant, except a significant interaction between history and environment for fecundity at generation 22 (see Table S3), with a steeper decline in the Warming environment for the populations of low latitude (see Fig. S1).

## Discussion

### Adaptation to a warming environment is slow

We observed a pattern of adaptation to a warming environment in *Drosophila subobscura* populations of southern (low latitude) European origin, with an improved reproductive success in the warming environment only detected after 39 generations of thermal evolution. Importantly, this improvement was not observed by generation 22, which also marks the end of the consistent increase in environmental warming across generations (that led to extremely low egg-to-adult viability as populations endured a daily peak temperature of 30 °C). Environmental temperature across generations remained unaltered from then onwards (until generation 39). This slow rate of thermal adaptation and the lack of response in the high latitude populations, calls into question the different potential of populations to show an effective adaptive response in the face of rapid environmental change.

There is a large body of experimental evidence highlighting constraints to evolution of upper thermal limits in physiological traits (see^6^ for a review), but fewer studies have tackled thermal limits for key life-history traits^42,43^. We provide further evidence for limits and historical contingencies of adaptation to changing thermal environments in an ectothermic species (see also below). To our knowledge, this is the longest experimental evolution study that analyses evolution under warming environments, incorporating increases in thermal amplitude across generations which are expected under predicted global warming scenarios^1^. In this vein, studies in environmental contexts that more closely match natural conditions such as mesocosms experiments will also be relevant in understanding species persistence under climate warming (e.g.,^44,45^).

Our findings complement those of a few other experimental studies covering shorter evolutionary time spans in highlighting the challenges and limits of thermal adaptive responses under ecological scenarios of environmental deterioration^14,18,20^. The fact that these studies were performed in a variety of ectotherm species suggests a fundamental limitation of adaptive evolution in face of rapid climate change regardless of the genetic composition of the populations. Constraints to the adaptive response under climate change might result from a generally lower heritability under warming conditions (e.g., see^46^ for an example). Nevertheless, in contrast to those studies, we show here that historically differentiated genetic backgrounds have an important impact in the evolutionary potential of populations.

Studies of evolutionary rescue of populations under deteriorating environments have highlighted the role of the rate of environmental change as critical in influencing the magnitude and efficacy of adaptive responses^3^, with slower rates of environmental change increasing the possibility for evolutionary rescue^47,48^. In our experiment, an adaptive response was only observed by generation 39 following a period where environmental change across generations was halted (from generation 22 onwards). On the other hand, no evolutionary response was observed by generation 22 following a temporal period of gradual environmental warming (between the start of the experiment and generation 22). This evidence suggests that adaptation in our populations was aided by the fact that the pace of environmental change was halted, although we cannot rule out the possibility that an adaptive response might have evolved shortly after generation 22—potentially resulting from the high selective pressures occurring towards the end of the period of gradual environmental change—as we did not further measure population response between generation 22 and generation 39. In any case, the rate of adaptation observed was quite low even if one considers that the later response occurred close to the first 22 generations of evolution. For sure slow adaptive rates will pose a severe constraint, and even mild-paced environmental changes might prove too big of a hurdle for populations to face. It is possible that a considerable fraction of the adaptive response comes from parental (transgenerational) effects due to continued exposure to the warming conditions (e.g.^14,49^). For instance, van Heerwarden and Sgrò^14^ found negative transgenerational effects during evolution of *D. melanogaster* and *D. pseudoananassae* under warming conditions. Additional experiments need to be performed to address such effects, namely studying adaptive responses following exposure of parental generation to distinct common garden environments (namely control and warming conditions).

### Reduced evidence for costs of adaptation across environments

Since our populations had already evolved for 70 generations in the ancestral, control environment, differences between the warming and control regimes in the latter environment were not expected unless costs of adaptation were involved. Trade-offs associated with adaptation to specific environments may occur due to antagonistic pleiotropy or linkage, with alleles having opposing fitness effects across environments^15^. In our experiment, we obtained evidence for an increased reproductive success and fecundity of the low latitude warming populations in their own environment, with some suggestion of lower performance in the control (ancestral) environment. This pattern suggests that adaptation to the warming conditions might have involved some costs, though not very robust ones. This is not surprising as costs of adaptation may be hard to track down and have seldom been found in the literature^50,51^.

### Inter-population variation in the adaptive response is context-dependent

As stated above, we found population-specific adaptive responses, with low latitude populations of the warming regime showing adaptation to the warming conditions while the same did not happen for the higher latitude populations which showed much higher heterogeneity in the response.

In a previous study in these populations, we showed variation in the shifts of thermal reaction norms after 31 generations of evolution in the warming environment, with high latitude populations from the warming regime increasing performance at higher temperatures^35^. Here, by testing the specific thermal conditions endured by our populations during their evolution, we found an opposite pattern: adaptation to warming conditions in populations of lower latitude, but not in the higher latitude ones. Possibly, the specificities of the different test environments—specific, constant temperatures in^35^ *versus* the exact environmental conditions of the selective regimes in the present study—promoted different outcomes in the two historically differentiated populations due to genotype by environment interactions. This study illustrates the importance of the details when testing for environment specific patterns of adaptation.

To our knowledge, no other experimental evolution studies have addressed variation in the adaptive response to warming conditions of populations with substantial contrasting past histories. In a plasticity experiment addressing the heat stress response in two *D. subobscura* populations subjected to stress during the development or adult stage, Porcelli et al.^29^ found evidence for a higher ability of low latitude populations in coping with heat stress than high latitude counterparts. While their results might sound to some extent connected with our findings, the fact is that our low latitude populations did not present a better performance than high latitude ones at the start of the study, only after evolving under thermal stress. This variation in the adaptive response might be due to a different pace of response between populations and/or to a higher depletion of genetic variation in the high latitude populations during thermal evolution. However, a more robust causal link between our results and the latitudinal variation in genetic backgrounds existent in nature would require sampling from the same as well as other natural locations, derived from additional latitudes.

We found context specific evolutionary potential to adapt to warming conditions in *D. suboscura* populations with contrasting past histories. The rate of adaptive response to the ecologically relevant thermal challenges was slow and only present in the populations of lower latitude. Altogether, our findings indicate that extended periods of evolution under environmental variation may be needed for an adaptive response to occur and that this response may vary at the inter-population level, highlighting important challenges for the ability of ectothermic populations to adapt to fast-paced environmental changes.

## Supplementary Information

Supplementary Information 1.

Supplementary Figure S1.

Supplementary Figure S2.

Supplementary Figure S3.

Supplementary Tables.

## Data Availability

Raw data is available in Supplementary Information.
