# Supplementary material for: Slow and population specific evolutionary response to a warming environment
Source: Sci Rep. 2023 Jun 15;13:9700. doi: 10.1038/s41598-023-36273-3 (PMC10272154; doi:10.1038/s41598-023-36273-3)
Supplement: Supplementary file 2 — Supplementary Figure S1. [file 41598_2023_36273_MOESM2_ESM.pdf]

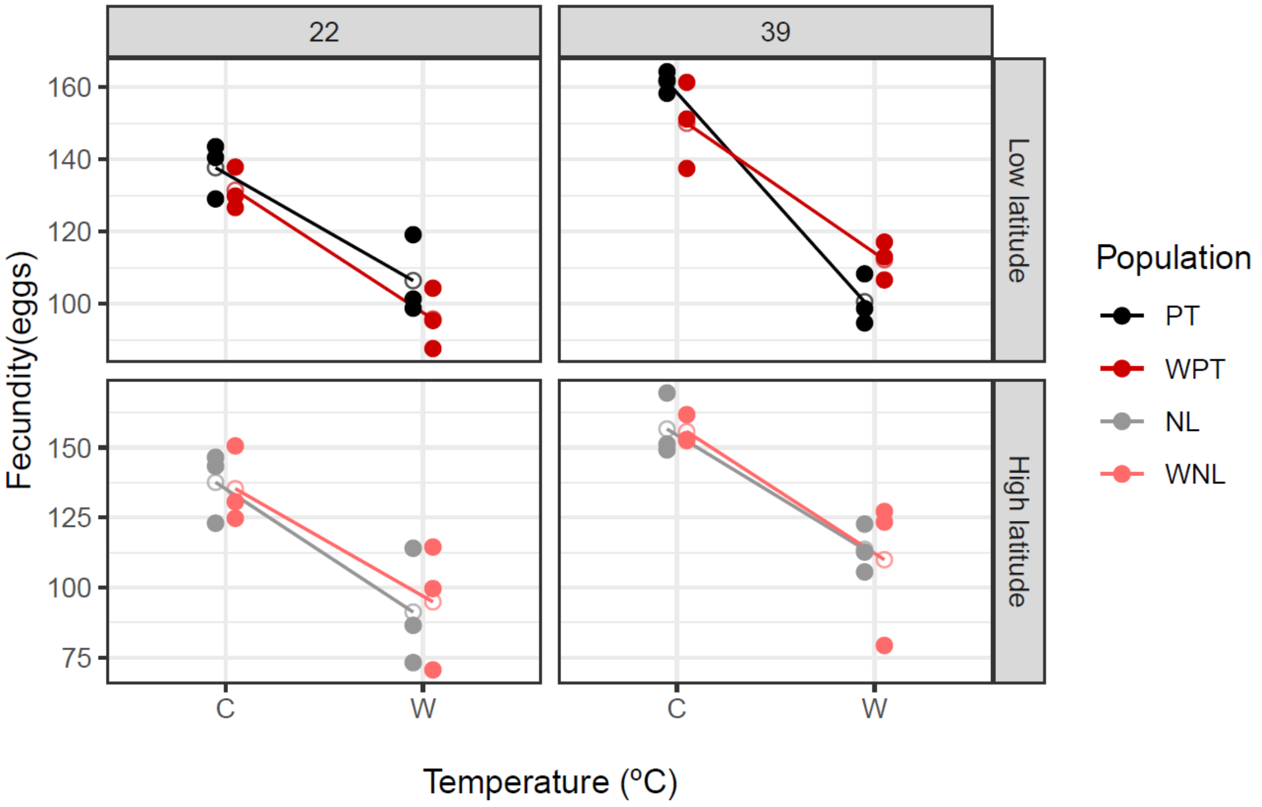

Supplementary Figure 1. Fecundity (number of eggs) of the warming (W) and control (C) populations in Warming and Control thermal environments at generations 22 and 39.
