# Supplementary material for: Slow and population specific evolutionary response to a warming environment
Source: Sci Rep. 2023 Jun 15;13:9700. doi: 10.1038/s41598-023-36273-3 (PMC10272154; doi:10.1038/s41598-023-36273-3)
Supplement: Supplementary file 3 — Supplementary Figure S2. [file 41598_2023_36273_MOESM3_ESM.pdf]

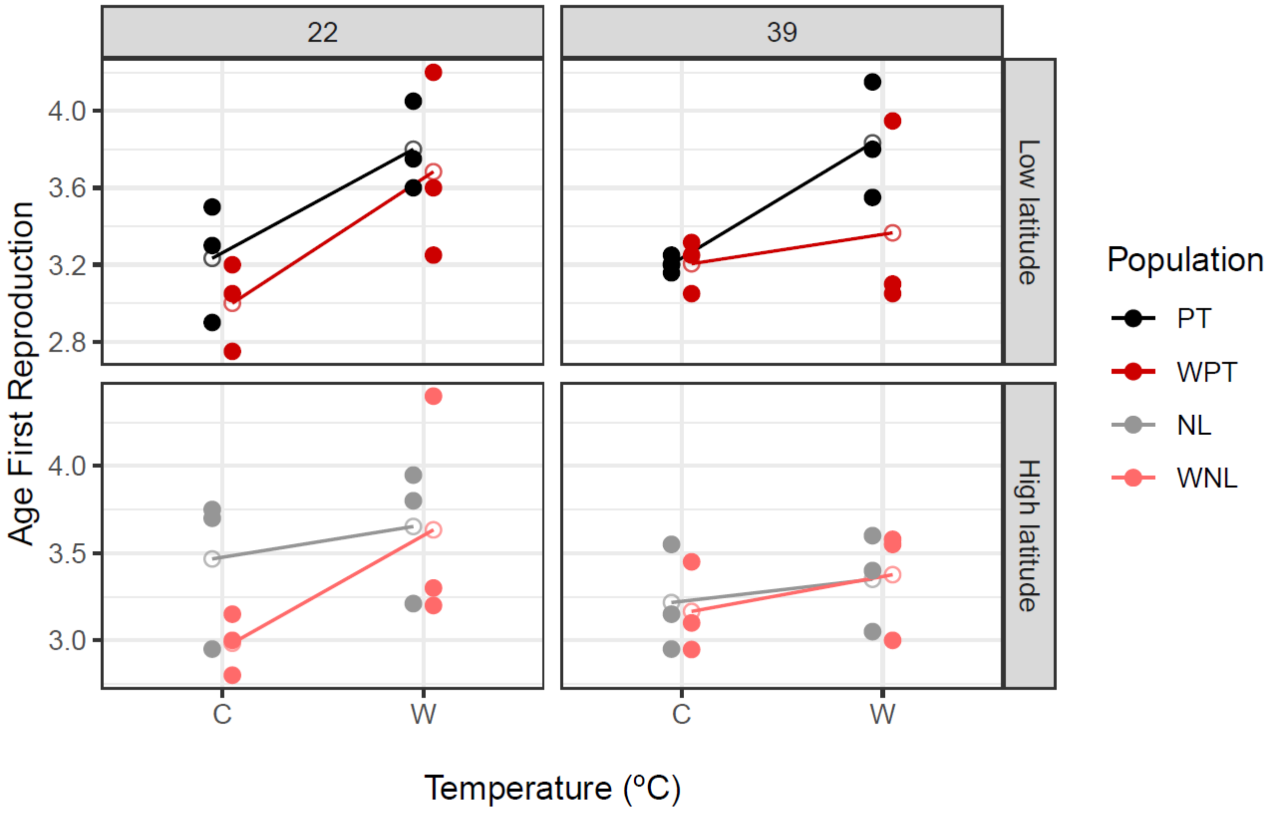

Supplementary Figure 2. Age of first reproduction (number of days) of the warming (W) and control (C) populations in Warming and Control thermal environments at generations 22 and 39.

-
